# Highly Selective and pH-Stable Reverse Osmosis Membranes Prepared via Layered Interfacial Polymerization

**DOI:** 10.3390/membranes12020156

**Published:** 2022-01-27

**Authors:** Min-Gyu Shin, Wansuk Choi, Jung-Hyun Lee

**Affiliations:** Department of Chemical and Biological Engineering, Korea University, 145 Anam-ro, Seongbuk-gu, Seoul 02841, Korea; aaas45@korea.ac.kr

**Keywords:** reverse osmosis, desalination, water treatment, thin film composite membrane, interfacial polymerization, layered interfacial polymerization, interlayer, pH stability

## Abstract

Ultrathin and smooth polyamide (PA) reverse osmosis (RO) membranes have attracted significant interest due to their potential advantages of high permeance and low fouling propensity. Although a layered interfacial polymerization (LIP) technique aided by the insertion of a polyelectrolyte interlayer has proven effective in fabricating ultrathin and uniform membranes, the RO performance and pH stability of the fabricated LIP membrane remain inadequate. In this study, a poly(piperazineamide) (PIPA) layer prepared via interfacial polymerization (IP) was employed as an interlayer to overcome the limitations of the prototype LIP method. Similar to the control polyelectrolyte-interlayered LIP membrane, the PIPA-interlayered LIP (pLIP) membrane had a much thinner (~20 nm) and smoother selective layer than the membrane fabricated via conventional IP due to the highly surface-confined and uniform LIP reaction. The pLIP membrane also exhibited RO performance exceeding that of the control LIP and conventional IP-assembled membranes, by enabling denser monomer deposition and a more confined interfacial reaction. Importantly, the chemically crosslinked PIPA interlayer endowed the pLIP membrane with higher pH stability than the control polyelectrolyte interlayer. The proposed strategy enables the fabrication of high-performance and pH-stable PA membranes using hydrophilic supports, which can be applied to other separation processes, including osmosis-driven separation and organic solvent filtration.

## 1. Introduction

Environmental pollution and freshwater shortages have become critical global challenges in modern society. To cope with these issues, technologies for producing clean water, including distillation, ion exchange, and membrane filtration, have been extensively developed and refined [1]. In particular, considerable advances in the reverse osmosis (RO) membrane process have secured its position as the most commonly used desalination technology, due to its low energy footprint and high-throughput productivity [2]. In the RO process, a semi-permeable RO membrane plays a pivotal role in water purification by discriminating dissolved salts from saline water under high-pressure conditions [3].

Conventional RO membranes have a thin-film-composite (TFC) structure composed of a porous polymeric support and a dense polyamide (PA) selective layer [4]. The PA selective layer is typically synthesized via interfacial polymerization (IP) of two monomers, *m*-phenylenediamine (MPD) and trimesoyl chloride (TMC), dissolved in immiscible water and an organic solvent, respectively [5,6]. This IP process enables the fabrication of TFC membranes with reliable RO performance in a scalable and fast manner. However, it produces an inherently heterogeneous, relatively thick, and rough PA structure with ridge-and-valley and/or nodular surface features [7], which hampers our fundamental understanding of the structure-performance relationship of the membrane. Furthermore, the inherent PA structure formed by conventional IP is unfavorable and impractical for achieving high water permeation and resisting membrane fouling [8,9,10]. Numerous efforts have been dedicated to the formation of a smooth, homogeneous, and ultrathin PA layer by employing new fabrication strategies, including molecular layer-by-layer [9,11], support-free IP [12,13], electrospraying [14,15], and dual-slot coating [16,17]. Although all the proposed strategies formed a smooth and ultrathin PA layer with good RO performance, they required labor-intensive protocols [18] and/or expensive experimental facilities [14,15].

Our research group has devised a new facile method to fabricate a smooth, uniform, and ultrathin PA layer, referred to as a layered IP (LIP) technique [19]. The LIP strategy relies on the pre-deposition of an electrostatic bilayer of oppositely charged polyelectrolytes (polyethyleneimine [PEI]/poly(acrylic acid) [PAA]) as an interlayer on a porous support. Subsequently, MPD monomers are uniformly and densely deposited on the interlayer in a highly surface-confined manner, followed by a crosslinking reaction with TMC, creating a PA permselective layer. The fabricated membrane (LIP membrane) exhibited a much smoother and thinner PA layer with improved RO performance compared to the membrane prepared via conventional IP (IP membrane) [19,20]. Despite its good RO performance, the NaCl rejection of the LIP membrane cannot meet the performance level of a commercial RO process (≥99.5%). Furthermore, the LIP membrane is prone to performance deterioration to pH changes due to the weak polyelectrolyte nature of its PEI/PAA interlayer, making the LIP technique commercially inviable [19].

In this study, we propose a new strategy to enhance the RO performance and pH stability of the LIP membrane by employing a poly(piperazineamide) (PIPA) layer, prepared via IP of piperazine (PIP) and TMC as an interlayer. PIPA chemistry has been widely used for nanofiltration membranes because of its high water permeance and good divalent salt rejection [21], which promotes its use as an interlayer with low hydrodynamic resistance. In addition, the PIPA layer has an intrinsically smooth surface with numerous surface carboxyl groups, which enables the uniform, compact, and strong MPD deposition [22], possibly constructing a smooth, dense, and robust PA layer via LIP. In particular, the crosslinked structure of the PIPA layer can also ensure good pH stability of the PIPA-interlayered LIP (pLIP) membrane. Despite its many potential advantages, the PIPA interlayer has not been used for membrane fabrication via LIP. The structure and performance of the pLIP membrane were optimized by tailoring both its PIPA interlayer and upper PA selective layer with varying monomer compositions. Furthermore, the structure, physicochemical properties, RO performance, and pH stability of the optimized pLIP membrane were comprehensively characterized and compared with those of the control LIP and conventional IP membranes.

## 2. Materials and Methods

### 2.1. Materials

PEI (*M_w_* = 50 kg mol^−1^, 50 wt.% in H_2_O, Sigma-Aldrich, St. Louis, MO, USA), PAA (*M_w_* = 100 kg mol^−1^, 35 wt.% in H_2_O, Sigma-Aldrich), PIP (99%, Sigma-Aldrich), MPD (99%, Sigma-Aldrich), TMC (>98.0%, TCI), hydrochloric acid (HCl, 35%, Daejung Chemical, Siheung, South Korea), NaCl (99.5%, Daejung Chemical), sodium hydroxide (NaOH, 98%, Daejung Chemical), *n*-hexane (99.5%, Daejung Chemical) were used as received. Deionized (DI) water (18.2 Ω) was prepared using a Millipore Milli-Q purification system. Polyacrylonitrile (PAN) ultrafiltration membranes (PAN50) were purchased from Nanostone Water (Waltham, MA, USA). Commercial RO membranes (SW30LE, SW30HR, BW30, and BW30LE from Dow Filmtec, Wilmington, DE, USA and SWC4+ from Hydranautics, Oceanside, CA, USA) were obtained from the manufactures.

### 2.2. Membrane Preparation

**Figure 1:**
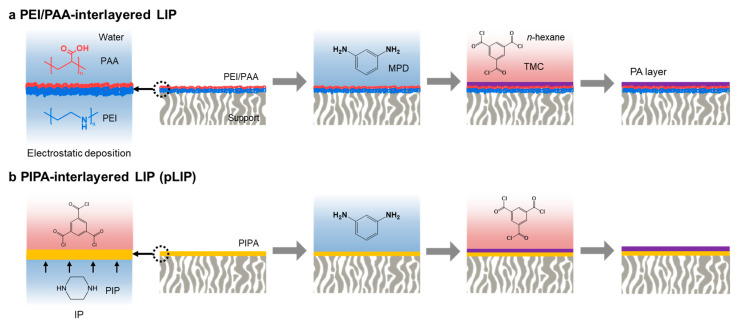
Schematic illustration of the membrane fabrication process. (**a**) Polyelectrolyte (PEI/PAA)-interlayered LIP and (**b**) poly(piperazineamide) (PIPA)-interlayered LIP (pLIP) membranes.

The TFC membrane was prepared by depositing the PEI/PAA or PIPA interlayer on a PAN support, followed by LIP (Figure 1). To fabricate the prototype PEI/PAA-interlayered LIP membrane (Figure 1a), the PAN support was first hydrolyzed by soaking it into a NaOH (1.5 M) aqueous solution at 45 °C for 120 min and subsequently washing it with DI water. This hydrolysis step enhanced the surface hydrophilicity and negative charge of the PAN support, thus facilitating the deposition of the PEI/PAA interlayer. Cationic PEI was electrostatically deposited on the hydrolyzed PAN (HPAN) support with an enhanced negative surface charge by immersing the support into a PEI (0.1 wt.%) aqueous solution containing 0.5 M NaCl (pH 10.6) for 15 min, followed by thorough rinsing with DI water. Subsequently, the support was immersed into an anionic PAA (0.1 wt.%) aqueous solution containing 0.5 M NaCl (pH 3.5) for 10 min and rinsed with DI water, producing the PEI/PAA polyelectrolyte interlayer. This interlayer blocked the support pores and thus enabled the effective construction of an upper PA selective layer via LIP [19]. The PA selective layer was fabricated on the PEI/PAA interlayer via LIP of MPD and TMC, following the protocol optimized in our previous report [19]. The PEI/PAA-interlayered HPAN support was dipped into an MPD (0.5 wt.%) aqueous solution for 3 min and then thoroughly rinsed with DI water. This rinsing step is a critical process in LIP. Rinsing removes loosely bound MPD, which allows for the surface-confined, uniform and tight MPD deposition, unlike conventional IP, where excess MPD impregnated in support pores is irregularly removed with a roller or an air knife. The support was then soaked into a TMC (0.1 wt.%) solution in *n*-hexane for 1 min to induce the crosslinking reaction between the pre-deposited MPD and TMC, forming a crosslinked, fully aromatic PA selective layer. After the LIP process, the membrane was rinsed with pure *n*-hexane and dried at 70 °C for 2 min.

To fabricate the pLIP membrane (Figure 1b), the HPAN support was dipped into a PIP (0.25–2.0 wt.%) aqueous solution for 3 min and then rinsed with DI water, followed by air gun blowing. Subsequently, the support was placed in contact with a TMC (0.0–1 wt.%) solution in *n*-hexane for 1 min, producing a PIPA interlayer. Next, the MPD/TMC-based PA selective layer was fabricated on the PIPA interlayer via LIP following the protocol described above using various MPD (0.05–5.0 wt.%) and TMC (0.01–0.5 wt.%) concentrations. It should be noted that the MPD adsorption time was fixed to 3 min because MPD adsorption for less than 3 min led to poor separation performance by lowering the concentration of adsorbed MPD.

For comparison, an IP membrane was also fabricated via conventional IP following the protocol optimized in our previous study [19]. The PAN support was impregnated with an MPD (0.5 wt.%) aqueous solution for 5 min, and then the excess MPD solution was removed with an air knife. Subsequently, the support was brought into contact with a TMC (0.1 wt.%) solution in *n*-hexane for 3 min, followed by rinsing with pure *n*-hexane and drying a 70 °C for 2 min.

### 2.3. Membrane Characterization

The surface morphologies of the membranes were examined using scanning electron microscopy (SEM, Inspect F50, FEI, Hillsboro, OR, USA) and atomic force microscopy (AFM, NX10, Park Systems, Suwon, Korea). The root-mean-square (*rms*) surface roughness of the membranes was quantified from their AFM topographic images of 5 × 5 μm^2^. Cross-sectional micrographs of the membranes were obtained using transmission electron microscopy (TEM, Titan TM 80-300, FEI, Hillsboro, OR, USA) at an accelerating voltage of 300 kV. The TEM sample was prepared by curing the membrane coupon in EPON™ resin, followed by slicing it into a nanometer-scale thickness using an ultramicrotome (Reichert Ultracut S, Leica, Wetzlar, Germany). Fourier transform infrared spectroscopy (FT-IR) and X-ray photoelectron spectroscopy (XPS) were used to analyze the chemical structures of the membranes. FT-IR was performed using a Spectrum Two spectrometer (PerkinElmer, Waltham, MA, USA) with an attenuated total reflectance unit. XPS spectra were collected on a PHI-5000 Versaprobe spectrometer (ULVAC-PHI, Chigasaki, Japan) using monochromatized Al–Kα radiation at 1486.6 eV. The water contact angle on the membrane surface was measured using a contact angle measurement system (Pheonix-300, SEO Corporation, Suwon, South Korea), and then converted to the solid–liquid interfacial free energy (−Δ*G*_SL_) to evaluate the intrinsic surface hydrophilicity of the membranes [23]. −Δ*G*_SL_ was calculated using the equation, −Δ*G*_SL_ = *γ_L_*(1 + cos*θ*/*r*), where *γ_L_* is the surface tension of water, *θ* is the measured water contact angle, and *r* is the ratio of the actual membrane surface area to the projected area determined using AFM [23]. The surface charge properties of the membranes were characterized by measuring their surface zeta potential at pH 5.8 using an electrophoretic measurement apparatus (ELS-2000Z, Otsuka Electronics, Hirakata, Japan).

### 2.4. Membrane Performance and pH Stability

The water flux (*J_w_*) and NaCl rejection (*R*_NaCl_) of the membranes were evaluated by permeating a NaCl (2000 ppm) aqueous solution across an effective membrane area (*a_m_*) of 14.5 cm^2^ in a cross-flow system at a pressure (Δ*P*) of 15.5 bar, a flow rate of 1 L min^−1^, and 25 °C. Performance data were collected after stabilization for 12 h. *J_w_* (L m^−2^ h^−1^, LMH) was calculated from the volume of the permeate (Δ*V*) collected for a certain time interval (*t*) using the equation, *J_w_* = Δ*V*/*a_m_t*. Water permeance (*A*, LMH bar^−1^) was then quantified using the equation, *J_w_*/(Δ*P* − Δ*π*), where Δ*π* is the transmembrane osmotic pressure difference [19]. *R*_NaCl_ (%) was calculated using the following equation, *R*_NaCl_ = 100 × (1 − *C_p_*/*C_f_*), where *C_p_* and *C_f_* are the NaCl concentrations of the permeate and feed, respectively [19]. The pH stability of the membranes was assessed by measuring their *A* and *R*_NaCl_ using feed solutions at different pH values, and the pH of each feed solution was adjusted using NaOH and HCl.

## 3. Results and Discussion

### 3.1. Membrane Performance

**Figure 2:**
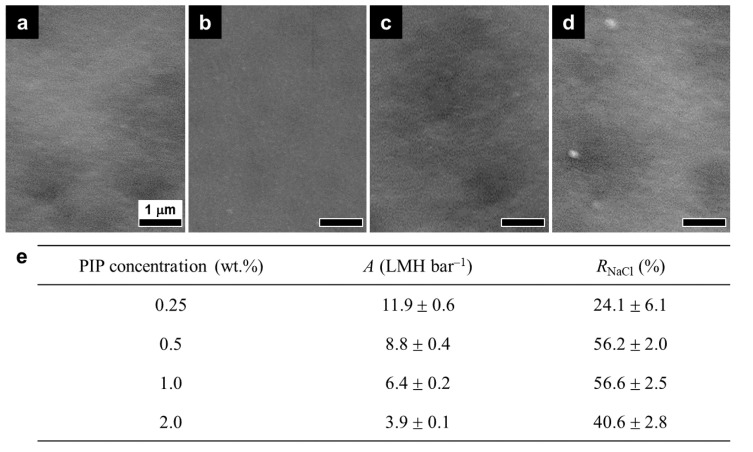
Surface morphologies and RO performance of the PIPA interlayers prepared with various PIP concentrations. (**a**–**d**) SEM surface images and (**e**) water permeance (*A*) and NaCl rejection (*R*_NaCl_) of the PIPA interlayers prepared at 0.1 wt.% TMC with different PIP concentrations: (**a**) 0.25, (**b**) 0.5, (**c**) 1.0, and (**d**) 2.0 wt.% PIP.

**Figure 3:**
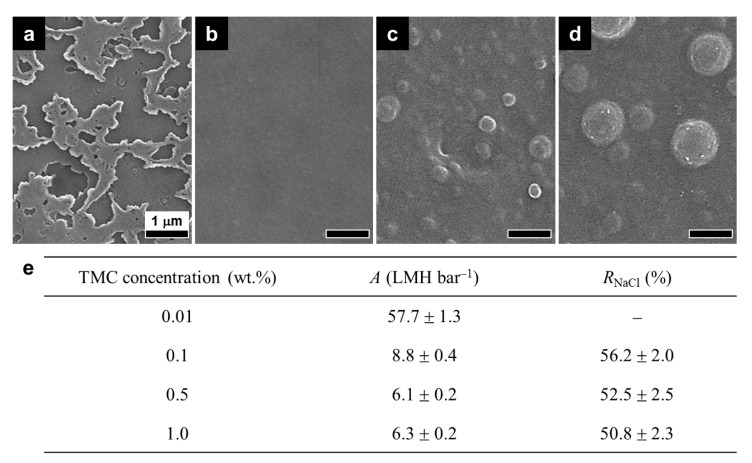
Surface morphologies and RO performance of the PIPA interlayers prepared with various TMC concentrations. (**a**–**d**) SEM surface images and (**e**) water permeance (*A*) and NaCl rejection (*R*_NaCl_) of the PIPA interlayers prepared at 0.5 wt.% PIP with different TMC concentrations: (**a**) 0.01, (**b**) 0.1, (**c**) 0.5, and (**d**) 1.0 wt.% TMC.

An optimal interlayer should have a structure that can effectively construct the upper PA selective layer while imparting minimal hydrodynamic resistance to the LIP-assembled membrane. Hence, the PIPA interlayer was optimized by characterizing its surface structure and RO performance as a function of PIP and TMC concentrations used for its formation (Figure 2 and Figure 3). At a fixed TMC concentration (0.1 wt.%), all the PIPA interlayers prepared with various PIP concentrations (0.25–2.0 wt.%) completely blocked support pores because no support pores were visible in SEM surface images (Figure 2a–d). Low PIP concentrations (0.25–1.0 wt.%) led to the formation of a uniform and smooth surface structure (Figure 2a–c), while the high PIP concentration (2.0 wt.%) produced some nodular surface features (Figure 2d). Along with the surface morphological change, increasing the PIP concentration progressively reduced *A* while increasing *R*_NaCl_ up to 1.0 wt.%, above which *R*_NaCl_ decreased (Figure 2e). A higher PIP concentration could increase the crosslinking density and thickness of the PIPA layer by enhancing the rate and extent of the IP reaction [24,25,26,27], resulting in a decrease in *A*, but an increase in *R*_NaCl_. However, excessive PIP concentrations (>1.0 wt.%) could induce the unbalanced stoichiometric reaction of PIP and TMC, yielding a looser, thicker, and irregular PIPA structure with loosely packed nodular surface features [25,28], which can account for the decline in both *A* and *R*_NaCl_ at 2.0 wt.% MPD.

At a fixed PIP concentration (0.5 wt.%), an exceedingly low TMC concentration (0.01 wt.%) produced a highly defective and irregular PIPA layer with remarkably high *A* but marginal *R*_NaCl_ (Figure 3a,e) due to the significantly suppressed IP reaction. The PIPA layer prepared with 0.1 wt.% TMC had a fairly smooth and uniform surface, exhibiting the highest *R*_NaCl_ and reasonably high *A* due to the optimized IP reaction (Figure 3b,e), indicating its suitability as a proper interlayer. At high TMC concentrations exceeding 0.1 wt.%, both *A* and *R*_NaCl_ of the PIPA layer decreased, and nodules were prominent on its surface (Figure 3c–e). This undesirable performance and surface structure of the PIPA layer could be attributed to the imbalanced stoichiometry of the PIP and TMC monomers that participated in the IP reaction, as mentioned above [22,29]. Based on these results, the optimal PIP and TMC concentrations were determined to be 0.5 and 0.1 wt.%, respectively, which produced a smooth, uniform, and defect-free PIPA layer with good permselectivity.

**Figure 4:**
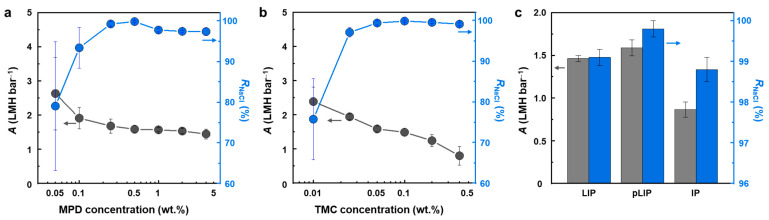
Performance optimization and comparison of the PIPA-interlayered LIP (pLIP) membranes. (**a**,**b**) Water permeance (*A*) and NaCl rejection (*R*_NaCl_) of the pLIP membranes prepared with (**a**) different MPD concentrations at 0.1 wt.% TMC and (**b**) different TMC concentrations at 0.5 wt.% MPD. (**c**) The RO performance of the optimized LIP, pLIP, and conventional IP membranes.

Following the optimization of the PIPA interlayer, the RO performance of the LIP-assembled PA selective layer was optimized by varying MPD and TMC concentrations (Figure 4). Like the PIPA interlayer, the balanced stoichiometry of MPD and TMC resulted in the optimal performance of the fabricated pLIP membrane (Figure 4a,b). Specifically, at a fixed TMC concentration (0.1 wt.%), low MPD concentrations (<0.5 wt.%) led to slightly higher *A* but relatively lower *R*_NaCl_ (Figure 4a), presumably due to the formation of a looser and thinner PA layer resulting from the lack of MPD monomers used for PA formation [30]. High MPD concentrations (>0.5 wt.%) also resulted in unsatisfactory RO performance (i.e., relatively low *A* and *R*_NaCl_). The excessive amount of MPD, which generated an improper MPD/TMC stoichiometry, could create a less crosslinked incipient PA layer, allowing MPD to continue to diffuse and react with TMC, consequently producing a less permselective PA layer [6,29]. At 0.5 wt.% MPD, the pLIP membrane exhibited the highest *R*_NaCl_ and satisfactory *A*, presumably due to the balanced MPD/TMC concentration.

On the other hand, at a fixed MPD concentration (0.5 wt.%), *A* decreased continuously with an increase in TMC concentration, while *R*_NaCl_ initially increased and then plateaued (Figure 4b). Higher TMC concentrations constructed a denser and thicker PA layer by facilitating the LIP reaction between MPD and TMC [30], thus yielding a more selective but less permeable membrane. The optimal MPD and TMC concentrations that achieved the best RO performance were 0.5 and 0.1 wt.%, respectively.

The RO performance of the optimized pLIP membrane was compared with that of the control LIP and IP membranes (Figure 4c). The conventional IP membrane exhibited poor RO performance with low values of *R*_NaCl_ (98.8 ± 0.3%) and *A* (0.86 ± 0.08 LMH bar^−1^). A conventional IP process is known to be inadequate for fabricating a highly permselective PA layer on a hydrophilic support such as PAN because the MPD solution forms a concave meniscus in the support pores and MPD diffusion is hindered by the support [26,31,32]. Both the LIP and pLIP membranes exhibited higher RO performance (i.e., higher *R*_NaCl_ and *A*) than the IP membrane, demonstrating that the LIP method is effective in fabricating a high-performance PA layer on a hydrophilic support. This is presumably because the LIP method can induce uniform and dense monomer deposition and generate a highly surface-confined reaction, enabled by the pre-deposition of the interlayer [19,26]. From a practical perspective, the LIP technique can broaden the application spectrum of PA TFC membranes to osmosis-driven separation (e.g., forward osmosis) and organic solvent filtration by fabricating hydrophilic support-based, high-performance membranes [33,34]. Importantly, the pLIP membrane (99.8 ± 0.2%, 1.59 ± 0.09 LMH bar^−1^) had higher *R*_NaCl_ and *A* values than the LIP membrane (99.1 ± 0.2%, 1.46 ± 0.04 LMH bar^−1^), meeting the *R*_NaCl_ level required for a commercial RO process (>99.5%). The superior performance of the pLIP membrane can be attributed to the advantageous attributes of the PIPA interlayer over PEI/PAA. Unlike the electrostatically assembled PEI/PAA interlayer that can absorb a certain amount of MPD, the tight molecular structure of the chemically crosslinked PIPA interlayer can effectively limit MPD deposition to its surface. As a result, the IP reaction could be more concentrated and confined to the interlayer surface of the pLIP membrane, inducing a more localized and denser interfacial reaction. Furthermore, the higher molecular density of the PIPA interlayer, as evidenced by its higher NaCl rejection than the PEI/PAA interlayer [19], possibly enriched surface carboxyl and amide groups, which would increase the number of deposited MPD monomers that would subsequently react with TMC, consequently forming a denser and more permselective PA layer. In particular, when compared with commercial and other reported lab-made RO membranes, the pLIP membrane exhibited moderate *A* but higher *R*_NaCl_ values (Table 1), highlighting its excellent and competitive RO performance.

**Figure 5:**
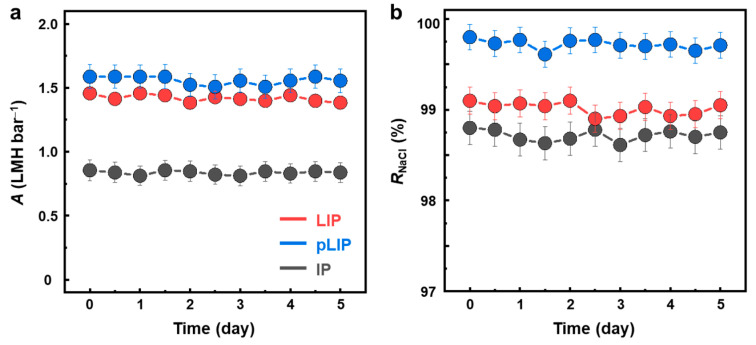
Long-term operational stability of the fabricated membranes. (**a**) Water permeance (*A*) and (**b**) NaCl rejection (*R*_NaCl_) of the optimized LIP, pLIP, and conventional IP membranes for 5 days of RO operation.

To evaluate the structural robustness of the fabricated membranes, we monitored their RO performance for 5 days (Figure 5). The RO performance of all the membranes remained nearly unchanged during the long-term period of operation, confirming their good structural stability.

### 3.2. Membrane Structure and Properties

**Figure 6:**
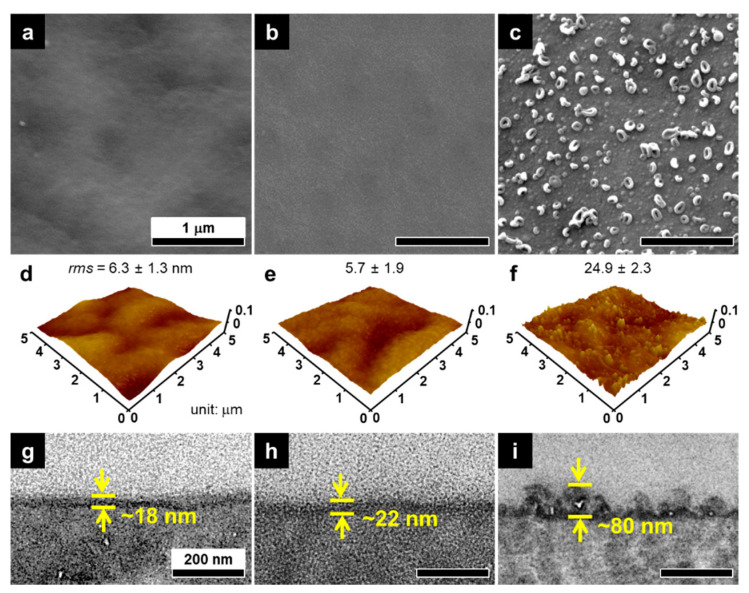
Morphologies of the fabricated membranes. (**a**–**c**) SEM surface, (**d**–**f**) AFM surface (with *rms* surface roughness), and (**g**–**i**) TEM cross-sectional images of the (**a**,**d**,**g**) LIP, (**b**,**e**,**h**) pLIP, and (**c**,**f**,**i**) conventional IP membranes.

**Figure 7:**
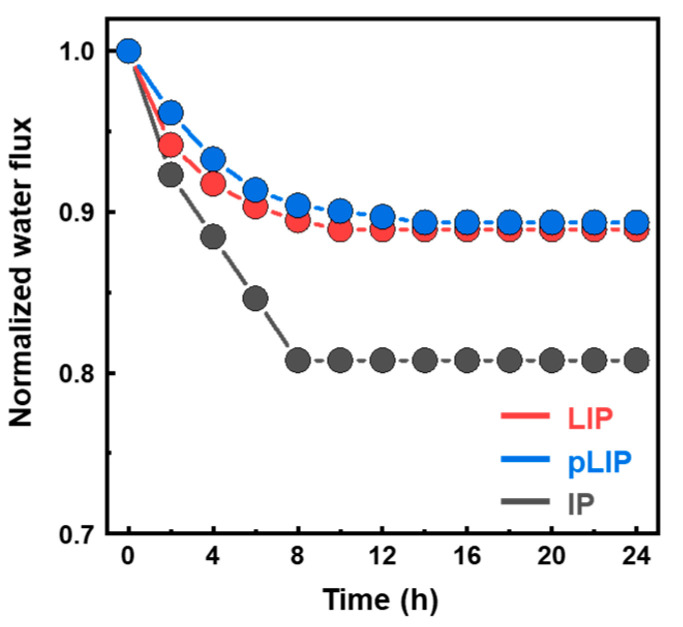
Water flux normalized by the initial value of the LIP, pLIP, and conventional IP membranes upon the addition of BSA to the DI water feed for 24 of RO operation.

The surface and cross-sectional morphologies of the optimized pLIP membrane were compared with those of the control LIP and IP membranes (Figure 6). The conventional IP membrane had a relatively thick (~80 nm) and rough (*rms* = ~25 nm) structure with typical ridge and nodular surface features (Figure 6c,f,i). In a typical IP process, excess MPD monomers impregnated in support pores vigorously and continuously diffuse to the organic phase and provoke a disordered and rapid crosslinking reaction with TMC [9,41,42], constructing an inherently heterogeneous and rough PA layer. Interestingly, both the LIP and pLIP membranes exhibited a much thinner (~20 nm), smoother (*rms* = ~6 nm), and more uniform selective layer structure than the conventional IP membrane. For the LIP and pLIP membranes, the presence of an interlayer could greatly confine the polymerization reaction zone near the interlayer surface by blocking support pores and thus allowing for MPD deposition on the interlayer surface [19,20,38,43]. Furthermore, the DI water rinsing step after MPD deposition could remove loosely bound MPD monomers, which enables minimal but uniform and dense MPD deposition, consequently creating an ultrathin and smooth PA layer after the reaction with TMC [19,20,38,43]. Despite its smoother surface with a less effective contact area compared with the IP membrane, the thinner selective layer structure of the LIP-assembled membrane could primarily contribute to its higher *A* by significantly reducing hydrodynamic resistance [44]. Together with excellent RO performance, the highly smooth surface of the LIP-assembled membrane would be beneficial for mitigating fouling by reducing the surface area for foulant attachment and accumulation [45]. To demonstrate this, we monitored the water flux of the fabricated membranes upon the addition of a model organic foulant (bovine serum albumin, BSA, 100 ppm) to the DI water feed for 24 h of RO operation (Figure 7). Both the LIP and pLIP membranes led to a low flux decline than the conventional IP membrane, confirming their better fouling resistance.

**Figure 8:**
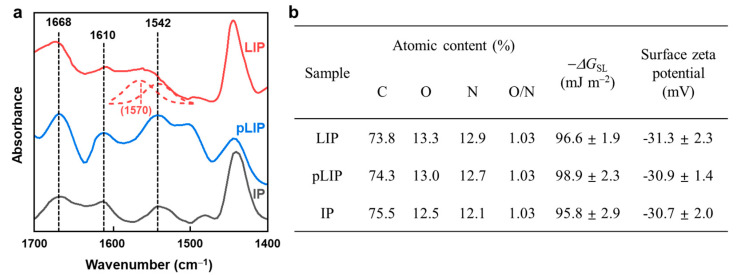
Physicochemical properties of the fabricated membranes. (**a**) FT-IR spectra and (**b**) XPS atomic content, intrinsic surface hydrophilicity (−Δ*G*_SL_), and surface zeta potential of the LIP, pLIP, and conventional IP membranes.

Figure 8 displays the physicochemical properties of the fabricated membranes. All the membranes showed the characteristic FT-IR peaks of MPD/TMC-based PA at 1668 cm^−1^ (C=O bond stretching), 1542 cm^−1^ (N–H bond in-plane bending), and 1610 cm^−1^ (H-bonded C=O bond stretching) (Figure 8a) [23,40], indicating the formation of a fully aromatic PA layer via IP or LIP. XPS analysis also revealed that the membranes had a nearly identical O/N ratio (~1.03), regardless of their fabrication methods (Figure 8b), confirming their similar chemical composition at the surface. Furthermore, there was no significant difference in the surface hydrophilicity (−Δ*G*_SL_) and charge between the membranes (Figure 8b). These results suggested that the excellent membrane performance of the LIP membrane was mainly due to its thin and dense structure rather than its physicochemical properties.

### 3.3. Membrane pH Stability

**Figure 9:**
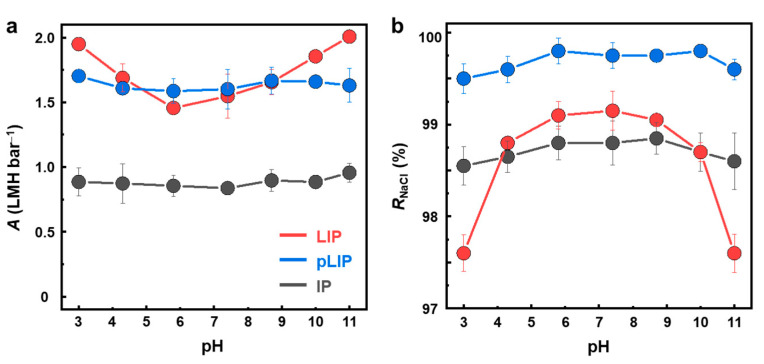
pH stability for the fabricated membranes. (**a**) Water permeance (*A*) and (**b**) NaCl rejection (*R*_NaCl_) of the LIP, pLIP, and conventional IP membranes as a function of the feed pH.

The pH stability of the membrane is an important consideration for its practical application, as the membrane is exposed to various pH conditions during cleaning and pre-treatment processes [46,47]. The prototype polyelectrolyte-interlayered LIP membrane lacks pH stability, possibly due to the weak polyelectrolyte nature of its PEI/PAA interlayer [19,48]. To demonstrate the enhanced pH stability of the newly developed pLIP membrane, its RO performance was monitored by varying the feed pH, and the results were compared with those of the control LIP and IP membranes (Figure 9). Unlike the conventional IP membrane exhibiting stable RO performance, regardless of the feed pH, the LIP membrane showed a noticeable reduction in *R*_NaCl_ with an increase in *A* under both acidic (pH < 6) and basic (pH > 8) conditions. This result indicated that the LIP membrane became defective under acidic and basic conditions, presumably due to the structural change in the PEI/PAA interlayer responding to pH conditions [49,50]. Specifically, since PEI tends to be protonated under acidic conditions, electrostatic repulsion between the PEI chains would be reinforced, inducing reorganization of polymer conformation [51]. Similarly, at higher pH, PAA becomes more anionic due to enhanced deprotonation, which would lead to significant electrostatic deformation of PAA [52]. As a result, the PEI/PAA interlayer could be more prone to structural disintegration when it was exposed to acidic or basic conditions, causing damage to the overlaid PA selective layer, which rendered the membrane less selective and more permeable. In contrast, the RO performance of the pLIP membrane remained intact over the entire pH range (3–11), demonstrating its superior pH stability to the polyelectrolyte-interlayered LIP membrane. The excellent pH stability of the pLIP membrane is likely due to its chemically crosslinked and pH-durable PIPA interlayer [24], which can ensure its stable operation under various pH conditions. Although most TFC RO membranes fabricated via conventional IP also perform stably in the pH range from 3 to 11, the pLIP membrane exhibited greater NaCl rejection and antifouling performance.

**Figure 10:**
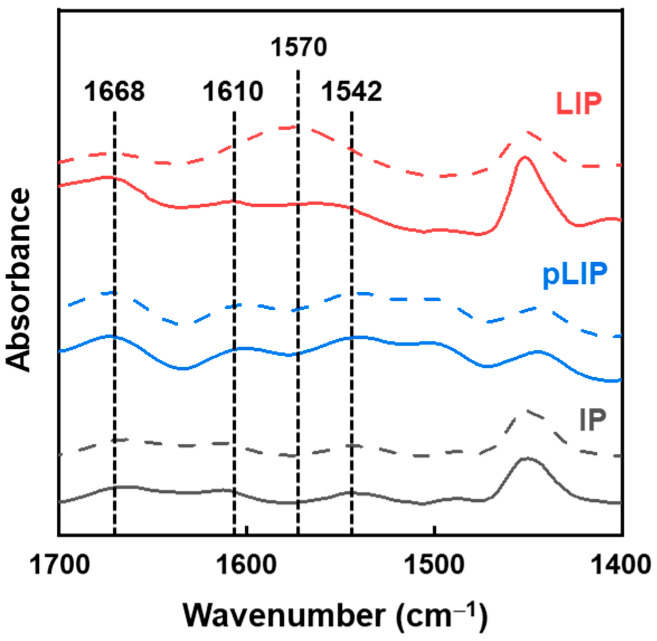
FT-IR spectra of the LIP, pLIP, and conventional IP membranes before (solid line) and after (dashed line) exposure to the acidic (2 h)/basic (2 h) condition.

Our claim was further confirmed by the change in the FT-IR spectra of the fabricated membranes after exposure to the acidic (pH 3) solution for 2 h, followed by the exposure to the basic solution for 2 h, as shown in Figure 10. Both the pLIP and conventional IP membranes exhibited the unchanged FT-IR spectra, even after exposure to the acidic/basic condition, which was consistent with their excellent pH stability. In contrast, the polyelectrolyte-interlayered LIP membrane showed the pronounced peak at 1570 cm^−1^ (asymmetric COO^−^ stretching), characteristic of the HPAN support [19], after exposure to the acidic/basic condition, which can be attributed to the destruction of the interlayer resulting from its poor pH stability.

## 4. Conclusions

In this study, a PA TFC membrane was prepared via LIP, where a PIP/TMC-based PIPA interlayer was introduced to improve the RO performance and pH stability of the prototype polyelectrolyte (PEI/PAA)-interlayered LIP membrane. Similar to the control LIP membrane, the PIPA-interlayered LIP (pLIP) membrane exhibited a much smoother and thinner selective layer structure than the conventional IP-assembled membrane by enabling confined, uniform, and dense MPD deposition and interfacial reaction. Although both the pLIP and control LIP membranes showed higher RO performance than the IP membrane, the pLIP membrane outperformed the LIP membrane and commercial RO membranes. The improved performance was related to the PIPA interlayer, which enabled denser MPD deposition and a more confined reaction than the PEI/PAA interlayer. Importantly, the chemically crosslinked and pH-stable nature of the PIPA interlayer resulted in the superior pH stability of the pLIP membrane compared to the PEI/PAA-interlayered LIP membrane. The proposed strategy can widen the application range of TFC membranes by achieving their high separation performance and pH durability using hydrophilic supports.

## Figures and Tables

**Table 1:** Performance comparison of the membranes. Water permeance (*A*) and NaCl rejection (*R*_NaCl_) of commercial and other reported lab-made RO membranes. The reported performance of lab-made membranes was measured under same operating conditions (feed concentration = 2000 ppm NaCl, operating pressure = 15.5 bar).

Membrane		*A* (LMH bar^−1^)	*R*_NaCl_ (%)	Reference
Commercial	SW30LE	1.11 ± 0.11	98.8 ± 0.1	This study
	SW30HR	1.03 ± 0.41	97.6 ± 1.1	
	SWC4+	1.44 ± 0.23	99.6 ± 0.5	
	BW30	2.93 ± 0.21	98.4 ± 0.3	
	BW30LE	3.76 ± 0.63	97.3 ± 0.5	
Lab-made		1.56 ± 0.24	98.7 ± 0.5	[19]
		1.60 ± 0.10	99.6 ± 0.3	[35]
		1.85 ± 0.08	99.3 ± 0.2	[36]
		3.74	98.3	[37]
		1.43 ± 0.08	99.0 ± 0.3	[38]
		2.74	98.2	[39]
		1.96 ± 0.14	99.5 ± 0.1	[40]
		1.59 ± 0.09	99.8 ± 0.2	This study (pLIP)

## Data Availability

The authors declare that all data supporting the findings of this study are available within the article.
